# A model based cost-utility analysis of Embedding referral to structured self-management education into standard practice (Embedding) compared to usual care for people with type 2 diabetes diagnosis in the last 12 months in England

**DOI:** 10.1136/bmjopen-2024-093327

**Published:** 2025-02-11

**Authors:** Daniel John Pollard, Anju Keetharuth, Alan Brennan, Danielle H Bodicoat, Agnieszka Glab, Michelle Hadjiconstantinou, Joesph P Mensa, Alison Northern, Melanie J Davies

**Affiliations:** 1 Sheffield Centre for Health And Related Research (SCHARR), School of Medicine and Population Health, The University of Sheffield, Sheffield, UK; 2 Independent Researcher, Leicester, UK; 3 Leicester Diabetes Centre, University Hospitals of Leicester NHS Trust, Leicester General Hospital, Leicester, UK; 4 Diabetes Research Centre, University of Leicester, Leicester, UK; 5 NIHR Leicester Biomedical Research Centre, Leicester General Hospital, Leicester, UK

**Keywords:** Diabetes Mellitus, Type 2, HEALTH ECONOMICS, Primary Care, Health Education

## Abstract

**Objectives:**

To conduct a cost-utility analysis of an implementation package that has been developed aiming to embed the referral of people with type 2 diabetes mellitus (T2DM) to structured self-management education (SSME) from primary care into routine practice compared with usual care.

**Design:**

Model-based cost-effectiveness analysis using the School for Public Health Research type 2 diabetes treatment model. With costs and effectiveness parameters coming from analyses of data from a cluster randomised control trial.

**Setting:**

English National Health Service.

**Participants:**

People with T2DM from 64 GP practices in England.

**Interventions:**

Embedding SSME implementation package Usual care.

**Primary and secondary outcome measures:**

The primary outcome measure was the incremental cost-effectiveness ratio. Secondary outcome measures included the probability of Embedding implementation package being cost-effective and value of information.

**Results:**

The estimated cost of the intervention was £40 316 across the study sites, which equates to £0.521 per patient across all practices. For the base case, the estimated mean discounted incremental lifetime cost of the intervention per patient is £48.19. This is associated with a mean per patient incremental quality-adjusted life-year (QALY) estimate of 0.006, producing an incremental cost-effectiveness ratio of £8311 per QALY gained. This has a 73.1% probability of the intervention being cost-effective at a funding threshold of £20 000 per QALY gained. Scenario analyses indicate that alternative parameterisations can lead to this finding being overturned.

**Conclusions:**

The effectiveness of the Embedding packages was hampered by the COVID-19 pandemic. However, our base case analysis shows that Embedding could be cost-effective for this patient population, but this was subject to significant structural uncertainty. This suggests that while implementation initiatives can be highly cost-effective in this population, more robust evidence or further incentivisation will be required before widespread adoption can be recommended.

**Trial registration number:**

ISRCTN23474120, registered 05/04/2018.

**STRENGTHS AND LIMITATIONS OF THIS STUDY**

- Data from the Embedding RCT has been used in an existing validated economic model for people type two diabetes to conduct a cost-utility analysis of the Embedding package compared with usual in the English National Health Service.
- The robustness of these results is tested in many scenario analyses.
- The Embedding RCT itself was disrupted by COVID-19, impacting the data that are included in these analyses.
- The 24 month RCT results, which drive the base case economic results, were statistically significant but the effects were small whereas the primary outcome from the 12 month data was not statistically significant meaning that the observed effects may reflect type 1 statistical error.
- We could only conduct scenario analyses on the duration of effects in the Embedding RCT, as the wait list design precluded direct estimation of duration effect.

## Introduction

 Type 2 diabetes mellitus (T2DM) generates a significant health and economic burden in the UK^1^ and globally.^2^ While the options for pharmacological treatment have broadened in recent years, there has also been a growing recognition of the importance of self-management. In response to this, several structured self-management education (SSME) packages, also known as diabetes self-management education and support (DSMES) programmes, have been developed and these have been shown to be associated with improvements in health outcomes.^3 4^ Furthermore, economic evaluations have shown SSME to be highly cost-effective.^5^,^7^

Despite this strong evidence of effectiveness and cost-effectiveness and 63.8% of people being offered an SSME package, reported uptake in England has been low with only 5.6% of people with T2DM recorded as attending SSME within 12 months of diagnosis.^8^ Among those that could attend, an important barrier to higher uptake was the lack of patient information relating to the benefits of participation, or ineffective communication of that information to people with T2DM.^9^ Based on these findings, a package of initiatives was developed to improve the uptake of SSME in UK primary care.^10^ The package had multiple components focussing on embedding referral to SSME into normal practice, with an emphasis on effective communication with patients and streamlining working practices for National Health Service (NHS) staff working with PWT2D.^10^

This ‘Embedding package’ was evaluated in a wait-list cluster randomised controlled trial (RCT) across a sample of UK general practices. The RCT found no statistically significant difference in the primary outcome measure of glycated haemoglobin (HbA1c) at 12 months between the intervention and control periods.^11^ However, an economic evaluation of the intervention using 24 month data was planned in order to assess the costs and effect of any longer-term changes associated with the package. A further benefit of this analysis is that the first year of the trial was seriously disrupted by the COVID-19 pandemic, and so the 24 month data may represent a more realistic picture of the package’s effectiveness.

In this paper, we report an economic evaluation based on the Embedding RCT, which calculates intervention costs for each practice, then combines these with the observed 24 month effects, which are then used to predict long-term sequelae of T2DM using the NIHR School for Public Health Research (SPHR) Type 2 Diabetes Treatment Model version 3.^12^

## Research design and methods

The Embedding study, including the intervention, is described in detail elsewhere,^11^ but in summary, 64 practices were randomised to either (A) a control wait-list group that did not receive any SSME referral support for 9 months before receiving the Embedding package for 9 months, or (B) an immediate intervention group that received the Embedding package for 18 months. Four SSME programmes were used: DESMOND (https://www.desmond.nhs.uk/), Diabetes 2gether/Diabetes 4ward (https://www.oxfordhealth.nhs.uk/community-diabetes/education/), Spotlight (Lincolnshire Community Health Services NHS Trust, terminated) and Xpert Health (https://www.xperthealth.org.uk/).

The Embedding package comprised the following key components: a marketing strategy to increase the level of awareness for SSME through GP practices and at specific events, user-friendly and streamlined referral pathways to access SSMEs and, a toolkit of resources available on an online portal with supporting resources for people with T2DM, healthcare professionals and other stakeholders.^13^ NHS Staff, employed centrally, who were trained in the application of the Embedding package—henceforth, referred to as ‘Embedders’-supported practices, commissioners and SSME programme providers to implement the package across all 64 practices. In addition to the primary outcome measure of HbA1c changes, other clinical measures, together with data on SSME referrals and attendance, were also collected.

Purposive sampling was undertaken to ensure that a representative sample of practices was taken.^10^ Full details on the randomisation are provided in the main trial paper^11^; in brief, GP practices were randomised 1:1 to Embedding or Control after an initial 3 month period to collect baseline data, with randomisation stratified by clinical commission group which commissioned primary care providers at the time of randomisation. Full details on the sample size are provided in the main study paper^11^; in summary, the study had 90% power to detect a 1.1 mmol/mol (0.1%) difference in HbA1c. Sensitivity analyses found that the power would still be at least 80% if the difference in HbA1c fell to 0.7 mmol/mol (0.06%) or if the average cluster size exceeded 174 people.

The methods for economic evaluation are in line with those specified by the National Institute for Health and Care Excellence (NICE).^14^ The perspective is that of the NHS and personal social services. Short-term data from the RCT are incorporated into a long-term health economic model, the NIHR SPHR T2DM treatment model V. 3.0.^12^ The analysis uses a lifetime horizon with discounting and/or annuitisation of costs and quality-adjusted life years (QALYs) undertaken at 3.5% per annum. All costs are pounds sterling and are in 2019/20 prices. All methods are in line with the study protocol, and the analysis plan for the modelling is available as a supplementary material (online supplemental file 2).^15^

### Data

Data collection of the tasks and activities for the Embedding package was undertaken by the Embedders. They recorded the initiatives undertaken, together with dates and time taken. Data on travel, subsistence, development of an online platform and the production of marketing materials were taken from records of project expenditure. No data were collected, nor costs were calculated, for practices during their control phases.

These data then enabled analysis of the intervention cost. Unit costs for the Embedding package relate to salaries and sundry expenditures, with the former based on *Unit Costs of Health and Social Care*,^16^ and the latter based on actual expenditure in the trial. The one-off cost of the toolkit was annuitized, in advance, over 5 years. Cost data relating to the SSME courses and long-term complications are taken from other published sources (online supplemental table S1). All costs are reported at 2019/20 price levels. Costs from previous years were inflated using the Hospital and Community Health Services Pay and Prices Index (up until 2014/15) and the National Health Service Cost Inflation Index (NHSCII) pay and prices index (from 2015/16 onwards).^16^

The Embedding RCT also provided the effectiveness data on changes in HbA1c, body mass index, systolic blood pressure, high-density lipoprotein (HDL) cholesterol and low-density lipoprotein(LDL) cholesterol for year one.^11^ Table 2 shows that differential effects were small, with a difference between the arms for HbA1c of just −0.01% at 1 year, and −0.05% at 2 years. The OR for attending SSME education was 18% lower at 1 year, but 15% higher at 2 years (which are parameterised as natural logarithms of the ORs in the modelling and reported in online supplemental table S2).

Long-term sequelae of T2DM based on the values for HbA1c, BMI, HDL, LDL and age/sex were estimated using the NIHR SPHR T2DM treatment model version 3.0,^12^ which is an individual-level simulation of diabetes-related complications for people with T2DM. Version 1 of the model was reported in detail previously.^17^ This was updated for this trial by including risk factor progression and risk equations from the United Kingdom Prospective Diabetes Study (UKPDS) 90,^18^ together with updated costs of diabetes-related complications (online supplemental table S1). For year one in the model, we used the trial data relating to changes in HbA1c, body mass index, systolic blood pressure, HDL cholesterol and LDL cholesterol for year one. Year two effects for HbA1c were taken from the observational follow-up analysis of the intervention arm of the trial. For BMI, HDL and LDL effects, it was assumed that they were the same as year one because year two observational follow-up data were not collected. The effect of Embedding on HbA1c at 2 years was assumed to be maintained for one more year with it then entirely waning by the end of year 4.

### Patient and public involvement

Patient (people with T2DM) and public involvement was used in the design of the research twice daily and was embedded in the overall research programme including the design of the Embedding programme and the design of the Embedding RCT. Dissemination of programme findings comprised in-person and electronic approaches. Two public engagement events were held. Two dissemination talks were also held; one in-person (36 attendees; all Asian women) and one remotely (20 attendees).

### Analysis

The costs of Embedding activities were calculated across the 33 immediate and 31 wait-list practices, and the individual SSME providers (who serviced multiple practices). Mean cost per person with a diagnosis of T2DM was calculated, after correcting for the length of follow-up, which differed in the two study arms due to the wait-list design.

Mean lifetime costs and QALYs were generated by the long-term model, with incremental cost-effectiveness ratios (ICERs), calculated where appropriate. As the model has been shown to be non-linear, the means were calculated via the probabilistic sensitivity analysis (PSA). Bootstrapped trial estimates were plotted on a cost-effectiveness plane and the associated cost-effectiveness acceptability curves were generated.^19^ When interpreting absolute cost-effectiveness, a threshold value of £20 000 per QALY gained was used, which is at the lower end of NICE’s specified threshold range.^14^

PSA was undertaken with all model parameters being randomly sampled from their associated distributions. Using a threshold ICER of £20 000 per QALY gained, we established that simulating 2000 samples of 5000 patients (10 million simulations in total) was sufficient to generate robust estimates of uncertainty in cost and QALY differences.^20^ Alongside our base case, six further scenario analyses were also undertaken to assess the impact of uncertainties not captured by probability distributions of the individual parameters. These were:

- Scenario one explored the effect of assuming that observed non-significant outcome differences in the RCT (with p values≥0.05) signified zero change. This meant that only the impact on HbA1c and attendance had influenced results.
- Scenario two explored using the results of the clinical primary analyses, which focused on outcomes at the end of twelve months (as opposed to 24 months). The main changes that this introduces are a reduced and more uncertain reduction in HbA1c, together with reduced SSME attendance.
- Scenario three explored using literature-based estimates of SSME effectiveness which were applied to referrals,^21^ as opposed to the trial estimates relating to the embedding package. Based on the DESMOND follow-up study and the STENO II trial, it was further assumed that SSME effects lasted for 6 years, which then reduced linearly to zero at ten years.^22 23^
- Scenario four explored using the results of clinical primary analyses (as in Scenario 2), together with the use of literature-based estimates of SSME effectiveness (as in Scenario 3), as opposed to those directly observed in the trial.
- Scenario five examined the impact of the longer duration of effects than those in the primary analysis. Alternative durations of 5 years and ten years, each with no effect thereafter, were assessed.
- Scenario six examined the impact of longer duration of SSME effect for Scenario 3. Alternative durations of ten years (which were then reduced linearly to zero at fifteen years) and patient lifetime were assessed.
- Scenario seven changed the source of the effect of Embedding on 1 year HbA1c to come from sensitivity analyses in the main trial paper and forthcoming monograph: the complete-case population, only those who attended education, using data collected prior to February 2020, only using providers who offered the DESMOND SSME course, only using providers who offered the Diabetes 2gether/Diabetes 4ward SSME course, only using providers who offered the Spotlight SSME course, only using providers who offered the Xpert Health SSME course.
- Scenario eight changed the source of the effect of Embedding on 1 year HbA1c to come from sensitivity analyses in the main trial paper on population characteristics (white ethnicity, ethnic minority groups and baseline HbA1c≥47.5 mmol/mol) and used a corresponding subgroup of the simulated population for the economic model.

Finally, to assess the remaining uncertainty in cost-effectiveness after the RCT and the potential value of any further research, we undertook the expected value of information analyses. These analyses place a monetary value on resolving any remaining uncertainties through undertaking future research.^24^ We used the Sheffield Accelerated Value of Information^25^ tool to produce estimates for the value of collecting further data on all uncertain parameters as well as three different parameter subgroup combinations: all intervention effectiveness parameters, all cost parameters and all utility parameters.

## Results

Table 1 shows the estimated cost of the intervention was £40 316 across the study sites. The biggest component of the quality adjusted life-adjusted life cost was the cost of staff time of the Embedders (71% of the total cost), and specifically, their work with the SSME provider organisations. Input from primary care practice staff and provider organisation staff was relatively small, with non-staff expenditure making up 17% of costs. Immediate practices (ie, those who received the intervention first) had higher costs than wait-list practices due to their longer follow-up (204 days vs 134 days, respectively), but when scaled to the same longer duration, mean cost per patient for the ITT analysis was similar (£0.49 vs £0.56, respectively). The cost used in the cost-effectiveness modelling (£0.521) was that for the ITT across all practices. All other model parameters are summarised in the Appendix.

**Table 1:** Intervention costs across all practices based on intention-to-treat population when the intervention was being delivered

Type of cost	Wait-list £ (Column %)	Immediate £ (Column %)	Provider £ (Column %)	Total costs £ Column %)
Embedder direct costs	3650 (13%)	7746 (27%)	17 200 (60%)	28 596 (71%)
Practice costs (staff)	185 (11%)	1471 (89%)	N/A	1656 (4%)
Provider costs (staff)	N/A	N/A	3358 (100%)	3358 (8%)
Travel and subsistence	551 (13%)	3827 (87%)	N/A	4378 (11%)
Marketing materials costs	600 (47%)	687 (53%)	N/A	1288 (3%)
Toolkit costs	504 (48%)	537 (52%)	N/A	1041 (3%)
**Total costs (Row %)**	**5490(14%)**	**14 268(35%)**	**20 558(51%)**	**40 316(100%)**
**Cost per participant adjusted to 204 day period**	**0.49**	**0.56**		**0.521**

For the base case, the estimated mean discounted incremental lifetime costs of the intervention per person with T2DM was small, at £47.67 (table 2). The probability of each individual health condition associated with T2DM was similar for both arms of the trial (online supplemental table S3). This leads to a small mean per person incremental QALY estimate of 0.0058, producing an ICER of £8311 per QALY gained.

**Table 2:** Results for base case analysis of cost-effectiveness

Base case	Control	Intervention	Increment	Probability embedding is cost-effective at £20 000 per QALY gained
Intervention cost per participant	£0	£0.52	£0.52	
Discounted lifetime costs of diabetes treatments and complications	£33 856	£33 904	£47.67	
Total discounted lifetime costs	£33 856	£33 904	£48.19	
Life years lived	15.116	15.121	0.006	
Discounted lifetime QALYs	7.3762	7.3820	0.0058	
Incremental cost per QALY gained (ICER)	–	–	£8311	73.1%

ICER, incremental cost-effectiveness ratioQALYs, quality adjusted life years

The results for the scenario analyses indicate that, in general, the intervention maintains its cost-effectiveness when alternative parameterisations are used (table 3). In three scenarios (S4, S7e, S7g), Embedding was found not to be cost-effective. In S4, this was due to the scenario’s incorporation of reduced SSME attendance and the use of lower literature-based estimates of clinical effectiveness. In S7e and S7g, this was due to Embedding increasing the mean HbA1c in these two scenarios.

**Table 3:** Results for base case analysis of cost-effectiveness and comparison with scenarios using alternative sources of evidence or assumptions

Scenarios	Incremental discounted lifetimeCosts per participant	Incrementaldiscounted QALYs per participant	ICER	Probability embedding is cost-effective at £20 000 per QALY gained
Base case	£48.19	0.0058	£8311	73.1%
S1—no insignificant secondary outcomes	−£4.06	0.0014	Embedding Dominates	83.0%
S2—only use results from the main step-wedge	−£5.22	0.0048	Embedding Dominates	85.9%
S3—only use SSME uptake from the trial, apply SSME effectiveness from a meta-analysis	£1.62	0.0020	£819	63.5%
S4—S3+S4	£1.37	−0.0030	Control Dominates	10.9%
S5a—5 years of full effect	£41.64	0.0093	£4500	84.4%
S5b—10 years of full effect	£33.05	0.0156	£2118	89.8%
S6a—S4+SSME benefits last in full for 10 years and wane after 15 years	£3.07	0.0025	£1210	65.5%
S6b—S4+SSME benefits last in full for a lifetime	£2.69	0.0031	£872	67.9%
S7a—1 year HbA1c estimated in the complete cases	£41.98	0.0074	£5681	79.9%
S7b—1 year HbA1c estimated in the education attenders	£43.71	0.0069	£6290	76.7%
S7c—1 year HbA1c estimated in data collected up until February 2020	£56.38	0.0040	£14 193	57.9%
S7d—1 year HbA1c estimated in providers who offer the DESMOND SSME course	£43.14	0.0071	£6060	76.6%
S7e—1 year HbA1c estimated in providers who offer the Diabetes 2gether or Diabetes 4ward SSME courses	£74.97	−0.0009	Control Dominates	30.6%
S7f—1 year HbA1c estimated in providers who offer the Xpert Health SSME course	£51.83	0.0052	£9920	60.0%
S7g—1 year HbA1c estimated in providers who offer the Spotlight SSME course	£63.86	0.0021	£29 771	48.2%
S8a—1 year Hba1c & a population subgroup in a white population	£48.38	0.0057	£8444	71.5%
S8b—1 year Hba1c & a population subgroup in an ethnic minority group population	£30.86	0.0144	£2146	82.1%
S8c—1 year Hba1c & a population subgroup in people with a baseline HbA1c≥47.5 mmol/mol	£47.59	0.0060	£7913	71.8%

ICER, incremental cost-effectiveness ratio; QALYs, quality adjusted life years; S, ScenarioSSME, structured self-management education

**Figure 1:**
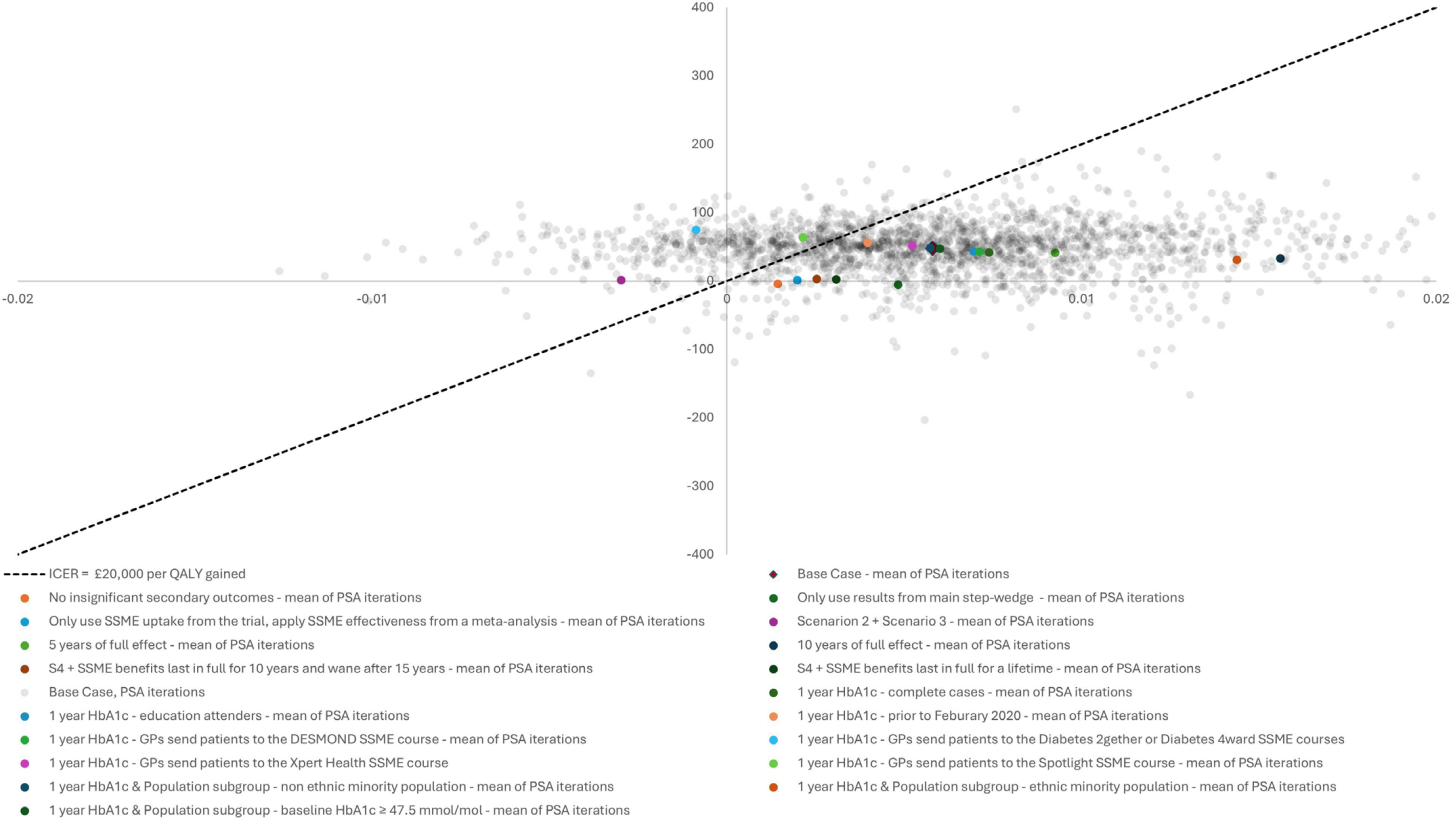
Cost-effectiveness plane for base case PSA and scenario analysis results.

Uncertainty analysis was undertaken using PSA, which shows samples present in all four quadrants of the cost-effectiveness plane (figure 1), that is, with these small effects on costs and on QALYs, we cannot be certain whether the intervention was providing positive or negative QALYs, or if it was cost saving or adding costs to the NHS. Overall, for the base case, there was a 73.1% probability of the intervention being cost-effective at a funding threshold of £20 000 per QALY gained. This ranges from around 60% to 90% in most of the scenarios examined. However S4, S7e and S7g which had a probabilities of Embedding being cost-effective of just 14.4%, 30.6% and 48.2% respectively (table 3). The base case PSA and the deterministic results of the scenario analyses were plotted on a cost-effectiveness plane and illustrate how many of the scenarios produce a reduced probability of the intervention being cost-effective (figure 1).

For the base case analysis, we also undertook value of information analysis to investigate whether it might be worth investing in further evidence collection to resolve remaining uncertainty after the trial. The estimated per person overall expected value of perfect information (EVPI) was small at just £15.25 per person (table 4). This would be the economic value of knowing for certain the exact value of every uncertain parameter in the cost-effectiveness model. We further calculated the value of information for subgroups of parameters. The three biggest subgroups of parameters contributing to uncertainty were those related to UKPDS risk equations for microvascular complications, UKPDS risk equations for macrovascular complications and the cost parameters in the model. Most importantly, we found that there was very little value in gathering further effectiveness data for the Embedding package, in fact the value was estimated at zero.

**Table 4:** Expected value of perfect information estimates for the base case cost-effectiveness model

Parameters	Per person EVPPI	Approx SE	Indexed to overall EVPI
Overall EVPI	15.25	NA	1.00
UKPDS OMv2: mortality equations	2.89	0.641	0.19
UKPDS OMv2: microvascular complication equations	4.50	0.696	0.29
UKPDS OMv2: macrovascular complication equations	3.64	0.654	0.24
UKPDS 90: risk factor progression and risk equations	2.06	0.673	0.14
Utility parameters	3.23	0.696	0.21
Cost parameters	3.61	0.662	0.24
Effectiveness parameters from Embedding	0.00	0.006	0.00

EVPI, expected value of perfect information; EVPPI, expected value of perfect parameter information; OMv2, United Kingdom Prospective Diabetes Study Outcome Model version 2

## Discussion

Our study describes the activities and costs associated with an implementation package that aimed to increase the uptake of SSME for people with T2DM who were under the care of a general practitioner (GP). The intervention package was found to have a low cost, with minimal additional costs being borne by the individual practices involved. However, only small overall effects were observed in relation to HbA1c and uptake at 24 months (which are contrary findings to those of the trial’s 12 month outcomes). When incorporated into a long-term cost-effectiveness simulation model, the base case results suggest that the Embedding intervention generates a small QALY gain, for a small additional cost, and has a 73.1% chance of being cost-effective. Our scenario analysis results suggest that there is significant structural uncertainty in this finding.

### Strengths and weaknesses

The strength of the trial is that it is based around a carefully developed implementation package that has been successfully tested,^10^ and that this targets changes in patient care and education that have been proven to improve diabetes control and associated health outcomes.^5 18^ Likewise, the long-term cost-effectiveness modelling is based on a previously validated model^17^ and is applied within a framework consistent with methods recommended by NICE.^14^

However, three issues are of particular note. First, the Embedding trial was disrupted by the COVID-19 pandemic. Embedding activities ceased part-way, when mainly the wait-list practices were meant to receive the intervention. The pandemic led to reduced SSME availability and, anecdotally, a reduction in the relative priority of SSME referrals by general practice staff, together with a reduction in the relative priority of SSME attendance by patients. Consequently, the effect size of the intervention is likely to have been diminished.

Second, while the 24 month results are statistically significant, the effects are small, and the primary outcome from the trial (at 12 months) was not statistically significant. In tandem with the low levels of implementation activities observed, as indicated by the low intervention costs shown in table 1, there is a concern that the observed effects may represent a type one statistical error. However, statistically significant improvements in HbA1c were found in ethnic minority subgroups.^11^ In addition, these results are also consistent with a lower-than-expected effect due to the COVID-19 pandemic and/or a slower-than-expected impact on referrals which is often seen with complex interventions.

Finally, there is uncertainty relating to the duration of the impact of the Embedding package as this could not be observed within the trial follow-up period, but we feel that the base case analysis adopts a conservative approach—that the duration of effect does not extend beyond 3 years. Less conservative assumptions examined in the scenarios all improve the estimate of cost-effectiveness.

### Implications for practice

The key question for practice is whether Embedding should be implemented/commissioned in the NHS. Our economic analyses show that Embedding could be cost-effective in most scenarios, which for most health economic studies would mean that the research team would be recommending implementation in clinical practice. However, we do not think that this is the case for Embedding for several reasons. First, as discussed above, there are substantial limitations to the evidence from the clinical study, especially because the SSME interventions were hugely affected by the COVID-19 pandemic. Second, the interpretation of the clinical trial results is less clear cut because it did not show any statistically significant effects on HbA1c or SSME uptake at 12 months in the whole study population, whereas improvements were observed in certain subgroups. This could lead clinicians and commissioners to conclude the intervention ‘does not work’. It did though produce effects that were statistically significant at 24 months, which may reflect that systemic changes take time to embed. Third, our expected value of information analysis around the base case indicates that the main sources of remaining decision uncertainty relate to the risk engine used for the long-term modelling, as opposed to the estimates of effectiveness. However, again this is nuanced because this ignores the uncertainties explored in the scenario analyses of table 3, which shows that different evidence or assumptions on the long-term effects could switch the decision conclusion from ‘Embedding dominates’ to ‘Control dominates’. All this taken together adds up to substantial uncertainty on whether we can and should recommend implementation of Embedding, based on the cost analysis. Since the trial, many of the good practices within the Embedding toolkit have been implemented by diabetes SSME providers, including wider adoption of self-referral and the use of social media, and such practices have been further adapted in line with the current ‘Digital First’ SSME landscape. However, there has been no move towards the adoption of a national/centralised Embedder role. Therefore, the research team’s considered judgement is that any similar (or dissimilar) approach to improve uptake of SSME should be tested and evaluated, ideally with a new randomised study, rather than just adopted by clinicians and commissioners.

### Further research

Our analysis shows that low-cost implementation interventions could be cost-effective for this population, even when the effect sizes are small and uncertain. While the value of collecting further information relating to the effectiveness of this package is small from a decision analytic perspective, this does not recognise uncertainties that are not captured by the parameterisation of the model base case (as exemplified by figure 1 or around limitations in the underlying key clinical study).^11^ Those uncertainties include possible alternative parameterisations captured by the scenario analyses, methodological uncertainties relating to the estimation of those parameters (eg, issues relating to the imputation of missing data, which were highlighted in the previously published clinical analysis), and limitations in the RCT itself.^11^ Consequently, for general practices or Integrated Care Boards to invest in interventions like the Embedding package, one might imagine they would prefer to see a more convincing set of outcome data, for example, larger effect sizes or significant changes across multiple outcome measures.

Alternatively, consideration should be given to greater incentivisation of referrals through the quality and outcomes framework (QOF). Currently, indicator DM014 links GP practice payments to the percentage of people newly diagnosed with diabetes who have a record of being referred to a SSME programme within 9 months after entry onto the diabetes register (https://www.england.nhs.uk/long-read/quality-and-outcomes-framework-guidance-for-2023-24/#section-2-summary-of-all-indicators4). Attaching a higher value to this indicator, more challenging thresholds for payment, or linking payments to attendance rather than referral to SSME may prove to be more successful, especially given the existence of a successfully tested Embedding package. The latter would also produce better quality information on attendance, which is currently recorded very poorly, and this would provide a more solid basis for future audits of SSME activity (and real-world effectiveness).

In conclusion, a novel implementation package was developed to facilitate the uptake of referrals to SSME and assessed within primary care GP practices in England. While the primary outcome of the trial failed to identify a significant effect, longer-term results indicate several changes that lead to the package being cost-effective. This incongruence of results and other uncertainties produced by the study, coinciding with the COVID-19 pandemic, suggest that while implementation initiatives can be highly cost-effective in this patient population more robust evidence or further incentivisation will be required before there is widespread adoption of this intervention.

## supplementary material

10.1136/bmjopen-2024-093327 online supplemental file 1

10.1136/bmjopen-2024-093327 online supplemental file 2

## Data Availability

Data are available in a public, open access repository.
