# Supplementary material for: A model based cost-utility analysis of Embedding referral to structured self-management education into standard practice (Embedding) compared to usual care for people with type 2 diabetes diagnosis in the last 12 months in England
Source: BMJ Open. 2025 Feb 11;15(2):e093327. doi: 10.1136/bmjopen-2024-093327 (PMC11815453; doi:10.1136/bmjopen-2024-093327)
Supplement: online supplemental file 1 [file bmjopen-15-2-s001.docx]

Contents

[Unit costs for costing the Embedding intervention and for long-term cost-effectiveness modelling 1](#_Toc175935607)

[Effectiveness Parameters 3](#_Toc175935608)

[Utility Parameters 7](#_Toc175935609)

[Risk equations for diabetes and weight related complications 9](#_Toc175935610)

[Risk factor Trajectories and risk equations for UKPDS 82 risk factors 9](#_Toc175935611)

[Health Economics and Decision Modelling Analysis Plan 14](#_Toc175935612)

[The probability of having different events in the model after 10 years 14](#_Toc175935613)

[References 14](#_Toc175935614)

# Unit costs for costing the Embedding intervention and for long-term cost-effectiveness modelling

| **Cost item** | **Cost (2019/20)**  **£** | | **Source** |
| --- | --- | --- | --- |
| **Embedding package** |  | |  |
| **Staff Costs (Total cost including in-costs per hour of staff contact time)** | | | |
| Embedder (Band 7 Agenda for Change band) | 58 | | PSSRU (2020)^1^ |
| Embedder (Band 5 Agenda for Change band) | 36 | |  |
| Practice administrative staff | 25 | |  |
| Practice manager | 58 | |  |
| Practice nurse | 39 | |  |
| Diabetes specialist nurse | 49 | |  |
| General practitioners | 156 | |  |
| Provider staff (lead) | 49 | |  |
| **Non-staff Costs** | | | |
| Marketing materials costs | £20 per practice | | Expenditure reports from within the trial. |
| Travel and subsistence expenses for Embedders | £18 per practice | |  |
| Website | £16 per practice | |  |
| **SSME course attendance** | | | |
| Cost per course per attendee | 94 | | Gillett (2010)^2^ |
| **Long-term annual costs for ongoing diabetes treatment** | | | |
| Metformin and primary care * | 634 | | Pollard (2023)^3^ |
| Metformin, sulfonylurea and primary care ** | 1095 | |  |
| Insulin and primary care*** | 2050 | |  |
| **Costs for fatal events predicted via modelling** | | | |
| Fatal myocardial infarction event | 586 | | Pollard (2023)^3^ |
| Fatal ischaemic heart disease event | 3067 | |  |
| Fatal stroke event | 3275 | |  |
| **Long-term annual costs for comorbidities predicted via modelling** | | | |
| Depression | 596 | | Pollard (2023)^3^ |
| Renal failure | 24040 | |  |
| Foot ulcer | 378 | |  |
| Cost per general practitioner visit | 39 | |  |
| Breast cancer | 12647 | |  |
| Colorectal cancer | 25362 | |  |
| Hypertension diagnosis | 44 | |  |
|  | **Year of event** | **Previous year** |  |
| Myocardial Infarction | 7010 | 922 |  |
| Stroke | 7632 | 984 |  |
| Chronic heart failure | 3514 | 1608 |  |
| Ischaemic heart disease | 10656 | 971 |  |
| Amputation | 12440 | 2666 |  |
| Blindness | 2381 | 221 |  |

| * | Metformin, 730 tablets per year, 1 Nurse appointment at the GP per year, 1 health care assistant appointment, 1 eye screening appointment, HbA1c, lipids, LFT, B12 and urine testing |
| --- | --- |
| ** | sitagliptin, 365 tablets per year, Metformin 730 tablets per year, Blood glucose testing (82.2 times per year), 1 Nurse appointment at the GP per year, 1 health care assistant appointment, 1 eye screening appointment, HbA1c, lipids, LFT, B12 and urine testing. |
| *** | Insulin glargine, 1 Nurse appointment at the GP per year, 1 health care assistant appointment, 1 eye screening appointment, HbA1c, lipids, lft B12 and urine testing. |

# Effectiveness Parameters

The base case effectiveness parameters were taken directly from the statistical analyses of the Embedding Study (reported in Davies *et al*.^4^)

To obtain the parameters for the scenario where the effectiveness of structured self management eduction (SSME) courses, we undertook a meta-analysis of SSME randomised controlled trials reported in Sargeant *et al*.^5^ This was a review in many different intervention types (including SSME), to establish how many people with an early diagnosis of T2D were included in large research trial. Data was extracted from the following studies and meta analysis of the summary results was conducted using Revman 5.

|  | Mean | Standard Error | Distribution | Source |
| --- | --- | --- | --- | --- |
| Embedding Study | | | | |
| Probability of SSME attendance in the control arm | 57.5% | NA | Beta(23376, 17243) | Davies *et al*.^4^ |
| One-year effects (Embedding v Control) | | | |  |
| HbA1c (%) | -0.01 | 0.01 | Normal | Davies *et al*.^4^ |
| BMI (Kg/m^2^) | -0.1 | 0.10 | Normal | Davies *et al*.^4^ |
| LDL cholesterol (mmol/L) | 0 | 0.01 | Normal | Davies *et al*.^4^ |
| HDL cholesterol (mmol/L) | 0 | 0.01 | Normal | Davies *et al*.^4^ |
| Systolic Blood Pressure (mmHg) | -0.3 | 0.22 | Normal | Davies *et al*.^4^ |
| Odds Ratio, SSME attendance | 0.82 | NA | Exponential^(Normal(-0.2,0.11) | Davies *et al*.^4^ |
| Two year effects (Embedding v Control) | | | | |
| HbA1c (%) | -0.05 | 0.01 | Normal | Davies *et al*.^4^ |
| Odds Ratio, SSME attendance | 1.15 |  | Exponential^(Normal(-0.14,0.05) | Davies *et al*.^4^ |
| Meta Analysis of SSME studies in Sargeant *et al*.^5^ (SSME v no SSME) | | | | |
| HbA1c (%) | -0.24 | 0.06 | Normal | De novo analysis |
| BMI (Kg/m^2^) | -0.33 | 0.22 | Normal | De novo analysis |
| LDL cholesterol (mmol/L) | -0.04 | 0.03 | Normal | De novo analysis |
| HDL cholesterol (mmol/L) | -0.01 | 0.02 | Normal | De novo analysis |
| Systolic Blood Pressure (mmHg) | -1.61 | 1.33 | Normal | De novo analysis |
| Scenario analyses on one-year effect on HbA1c (%) (Embedding v Control) | | | | |
| Complete case population | -0.14 | 0.16 | Normal | Davies *et al*.^4^ |
| Education attenders | -0.11 | 0.20 | Normal | Davies *et al*.^4^ |
| Data collected prior to February 2020 | +0.12 | 0.17 | Normal | Davies *et al*.^4^ |
| Providers offered DESMOND SSME course | -0.11 | 0.20 | Normal | Davies *et al*.^6^ |
| Providers offered Diabetes 2gether/ Diabetes 4ward SSME course | +0.48 | 0.32 | Normal | Davies *et al*.^6^ |
| Providers offered Spotlight SSME course | +0.25 | 0.43 | Normal | Davies *et al*.^6^ |
| Providers offered Xpert Health SSME course | -0.01 | 0.77 | Normal | Davies *et al*.^6^ |
| White ethnic subgroup | -0.02 | 0.16 | Normal | Davies *et al*.^4^ |
| Ethnic minorities subgroup | -0.64 | 0.22 | Normal | Davies *et al*.^4^ |
| Baseline HbA1c ≥ 47.5 mmol/mol | -0.04 | 0.19 | Normal | Davies *et al*.^4^ |
| NA, not applicable; SSME, structured self-management education | | | | |

Included studies in the meta analysis

| Sone 2002^7^ |
| --- |
| Young 2005^8^ |
| Deakin 2006^9^ |
| Adolfson 2007^10^ |
| Ko 2007^11^ |
| Davies 2008^12^ |
| Sturt 2008^13^ |
| Gary 2009^14^ |
| Trento 2010^15^ |
| Walker 2011^16^ |
| Ali 2016^17^ |
| Odoletkova 2016^18^ |
| Andrews 2011^19^ |

Meta analysis results

The random effects meta analysis of HbA1c at 1 year post treatment
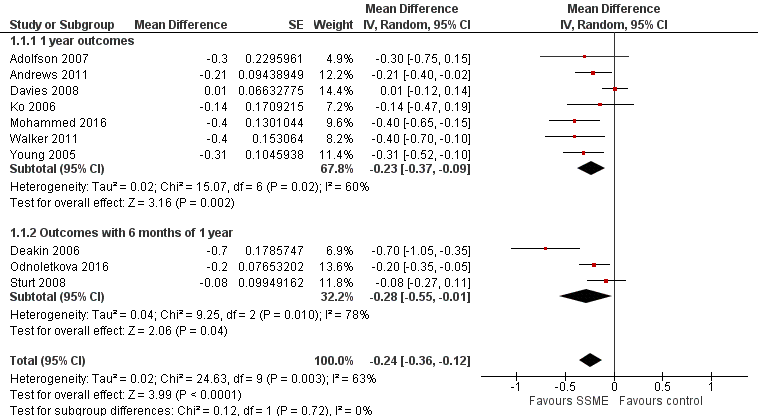


The random effects meta analysis of HbA1c at 4 years post treatment


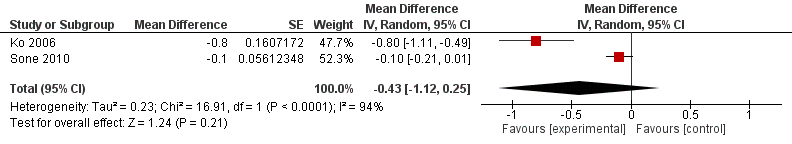


The random effects meta analysis of BMI at one year post-treatment


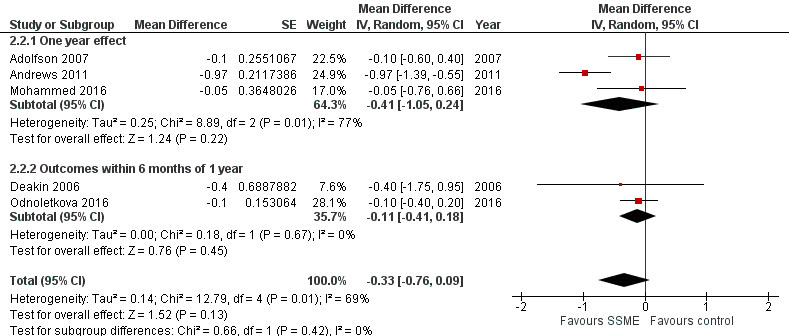


The random effects meta analysis of HDL at 1 year


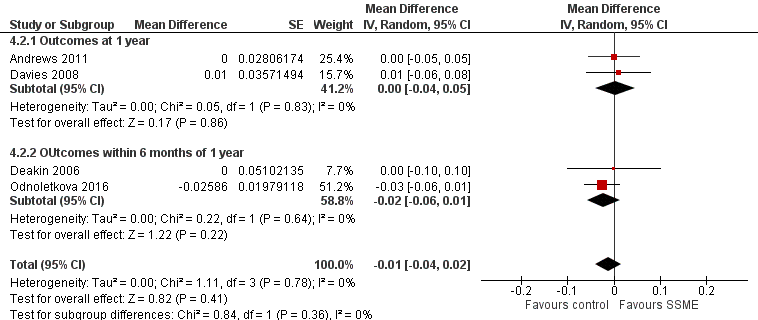


The random effects meta analysis of LDL cholesterol


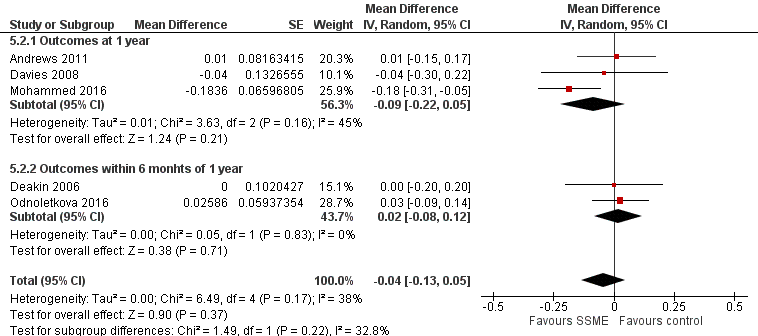


The random effects meta analysis of systolic blood pressure


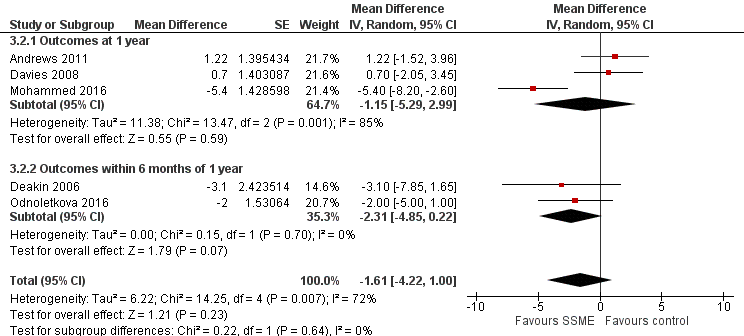


# Utility Parameters

Utility parameters used in the model

| **Utility parameters** | **Mean** | **SE** | **Source** |
| --- | --- | --- | --- |
| **Utility for someone with Type 2 Diabetes and no complications** | | | |
| Baseline utility | 0.83 | 0.0022 | Hayes et al 2016^20^ |
| Utility in an Age-gender matched population | 0.818 | NA | Hayes et al 2016^20^  Ara and Brazier^21^ |
| Multiplier | 1.01 | NA | Calculated |
| **Excess BMI** | | | |
| Decrement | 0.003 | 0.000 | Laxy et al. 2021^22^ |
| **Myocardial Infarction – Year of event** | | | |
| Decrement | 0.065 | 0.03 | Alva et al 2014^23^ |
| Without MI | 0.807 | 0.005 | Alva et al 2014^23^ |
| With MI | 0.742 | NA | Calculation |
| Multiplier | 0.919 | NA | Calculation |
| **Myocardial Infarction –event in a previous year** | | | |
| Additional Decrement | 0.008 | 0.024 | Alva et al 2014^23^ |
| Without MI | 0.807 | 0.005 | Alva et al 2014^23^ |
| With MI | 0.75 | NA | Calculation |
| Multiplier | 0.929 | NA | Calculation |
| **Stroke** | | | |
| Decrement | 0.099 | 0.035 | Hayes et al 2016^20^ |
| Without Stroke | 0.83 | 0.002 | Hayes et al 2016^20^ |
| With Stroke | 0.731 | NA | Calculation |
| Multiplier | 0.881 | NA | Calculation |
| **IHD** | | | |
| Decrement | 0.01 | 0.029 | Hayes et al 2016^20^ |
| Without IHD | 0.83 | 0.002 | Hayes et al 2016^20^ |
| With IHD | 0.82 | NA | Calculation |
| Multiplier | 0.988 | NA | Calculation |
| **CHF** | | | |
| Decrement | 0.045 | 0.040179 | Hayes et al 2016^20^ |
| Without IHD | 0.83 | 0.002186 | Hayes et al 2016^20^ |
| With IHD | 0.785 | NA | Calculation |
| Multiplier | 0.946 | NA | Calculation |
| **Blindness** | | | |
| Decrement | 0.033 | 0.027 | Alva et al 2014^23^ |
| Without Blindness | 0.807 | 0.005 | Alva et al 2014^23^ |
| With Blindness | 0.774 | NA | Calculation |
| Multiplier | 0.959 | NA | Calculation |
| **Foot Ulcer** | | | |
| Decrement | 0.17 | 0.019 | Bagust and Beale 2005^24^ |
| Without Foot Ulcer | 1.027 | 0.027 | Bagust and Beale 2005^24^ |
| With Foot Ulcer | 0.857 | NA | Calculation |
| Multiplier | 0.834 | NA | Calculation |
| **Amputation** | | | |
| Decrement | 0.172 | 0.045 | Alva et al 2014^23^ |
| Without Amputation | 0.807 | 0.005 | Alva et al 2014^23^ |
| With Amputation | 0.635 | NA | Calculation |
| Multiplier | 0.787 | NA | Calculation |
| **MMALB** | | | |
| Decrement | 0.048 | 0.022 | Bagust and Beale 2005^24^ |
| Without Foot Ulcer | 1.027 | 0.027 | Bagust and Beale 2005^24^ |
| With Foot Ulcer | 0.979 | NA | Calculation |
| Multiplier | 0.953 | NA | Calculation |
| **ESRD** | | | |
| Decrement | 0.049 | 0.068 | Hayes et al 2016^20^ |
| Without ESRD | 0.83 | 0.002 | Hayes et al 2016^20^ |
| With ESRD | 0.781 | NA | Calculation |
| Multiplier | 0.941 | NA | Calculation |
| **PVD** | | | |
| Decrement | 0.061 | 0.015 | Bagust and Beale 2005^24^ |
| Without PVD | 1.027 | 0.027 | Bagust and Beale 2005^24^ |
| With PVD | 0.966 | NA | Decrement |
| Multiplier | 0.941 | NA | Decrement |
| **ATFIB** | | | |
| Decrement | None | NA | No sources appear to have a decrement for this. |
| Multiplier | 1 | NA | Assumption |

# Risk equations for diabetes and weight related complications

The parameters used in the risk equations are detailed in full in sections 9.2 to 9.5 of Breeze *et al*.^25^

The risk equations for cardiovascular disease and mortality are taken from UKPDS 82.^26^ These are included in the model using the published means and standard errors.

# Risk factor Trajectories and risk equations for UKPDS 82 risk factors

All risk factor trajectories were taken from UKPDS 82 – for clarity the values and distributions assumed in the PSA are replicated here.

| Risk factor parameter | Mean | Standard Error | Distribution | Source |
| --- | --- | --- | --- | --- |
| HbA1c – panel data regression | | | | |
| Constant | 1.42 | NA | Bootstrapped Samples | UKPDS 90^27^ |
| Woman | 0.05 | NA | Bootstrapped Samples | UKPDS 90^27^ |
| Afro Caribbean Ethnicity | 0.07 | NA | Bootstrapped Samples | UKPDS 90^27^ |
| Indian Ethnicity | 0.05 | NA | Bootstrapped Samples | UKPDS 90^27^ |
| HbA1c last year | 0.72 | NA | Bootstrapped Samples | UKPDS 90^27^ |
| Natural logarithm (years since diagnosis) | 0.14 | NA | Bootstrapped Samples | UKPDS 90^27^ |
| HbA1c at diagnosis | 0.08 | NA | Bootstrapped Samples | UKPDS 90^27^ |
| BMI – panel data regression | | | | |
| Constant | 0.04 | NA | Bootstrapped Samples | UKPDS 90^27^ |
| Woman | -0.09 | NA | Bootstrapped Samples | UKPDS 90^27^ |
| Afro Caribbean Ethnicity | -0.09 | NA | Bootstrapped Samples | UKPDS 90^27^ |
| Indian Ethnicity | 0.95 | NA | Bootstrapped Samples | UKPDS 90^27^ |
| BMI last year | -0.16 | NA | Bootstrapped Samples | UKPDS 90^27^ |
| Natural logarithm (years since diagnosis) | 0.03 | NA | Bootstrapped Samples | UKPDS 90^27^ |
| BMI at diagnosis | 0.04 | NA | Bootstrapped Samples | UKPDS 90^27^ |
| LDL cholesterol – panel data regression | | | | |
| Constant | 0.76 | NA | Bootstrapped Samples | UKPDS 90^27^ |
| Woman | 0.06 | NA | Bootstrapped Samples | UKPDS 90^27^ |
| Afro Caribbean Ethnicity | -0.05 | NA | Bootstrapped Samples | UKPDS 90^27^ |
| Indian Ethnicity | -0.07 | NA | Bootstrapped Samples | UKPDS 90^27^ |
| LDL last year | 0.58 | NA | Bootstrapped Samples | UKPDS 90^27^ |
| Natural logarithm (years since diagnosis) | -0.04 | NA | Bootstrapped Samples | UKPDS 90^27^ |
| LDL at diagnosis | 0.21 | NA | Bootstrapped Samples | UKPDS 90^27^ |
| HDL cholesterol – panel data regression | | | | |
| Constant | 0.17 | NA | Bootstrapped Samples | UKPDS 90^27^ |
| Woman | 0.04 | NA | Bootstrapped Samples | UKPDS 90^27^ |
| Afro Caribbean Ethnicity | 0.05 | NA | Bootstrapped Samples | UKPDS 90^27^ |
| HDL last year | 0.60 | NA | Bootstrapped Samples | UKPDS 90^27^ |
| HDL at diagnosis | 0.22 | NA | Bootstrapped Samples | UKPDS 90^27^ |
| SBP – panel data regression | | | | |
| Constant | 29.01 | NA | Bootstrapped Samples | UKPDS 90^27^ |
| Woman | 0.68 | NA | Bootstrapped Samples | UKPDS 90^27^ |
| Indian Ethnicity | -1.39 | NA | Bootstrapped Samples | UKPDS 90^27^ |
| LDL last year | 0.67 | NA | Bootstrapped Samples | UKPDS 90^27^ |
| Natural logarithm (years since diagnosis) | 0.57 | NA | Bootstrapped Samples | UKPDS 90^27^ |
| LDL at diagnosis | 0.12 | NA | Bootstrapped Samples | UKPDS 90^27^ |
| Heart Rate (beats per minute) – panel data regression | | | | |
| Constant | 31.23 | NA | Bootstrapped Samples | UKPDS 90^27^ |
| Woman | 1.01 | NA | Bootstrapped Samples | UKPDS 90^27^ |
| Heart Rate last year | 0.33 | NA | Bootstrapped Samples | UKPDS 90^27^ |
| Natural logarithm (years since diagnosis) | 0.92 | NA | Bootstrapped Samples | UKPDS 90^27^ |
| Heart Rate at diagnosis | 0.27 | NA | Bootstrapped Samples | UKPDS 90^27^ |
| WBC – panel data regression | | | | |
| Constant | 1.45 | NA | Bootstrapped Samples | UKPDS 90^27^ |
| Woman | 0.09 | NA | Bootstrapped Samples | UKPDS 90^27^ |
| Indian Ethnicity | -0.33 | NA | Bootstrapped Samples | UKPDS 90^27^ |
| WBC last year | 0.46 | NA | Bootstrapped Samples | UKPDS 90^27^ |
| Natural logarithm (years since diagnosis) | 0.17 | NA | Bootstrapped Samples | UKPDS 90^27^ |
| WBC at diagnosis | 0.29 | NA | Bootstrapped Samples | UKPDS 90^27^ |
| Haemoglobin – panel data regression | | | | |
| Constant | 5.04 | NA | Bootstrapped Samples | UKPDS 90^27^ |
| Woman | -0.35 | NA | Bootstrapped Samples | UKPDS 90^27^ |
| Afro Caribbean Ethnicity | -0.19 | NA | Bootstrapped Samples | UKPDS 90^27^ |
| Natural logarithm (years since diagnosis) | -0.33 | NA | Bootstrapped Samples | UKPDS 90^27^ |
| Haemoglobin at diagnosis | 0.69 | NA | Bootstrapped Samples | UKPDS 90^27^ |
| Smoking status – logistic regression | | | | |
| Constant | 0.02 | NA | Bootstrapped Samples | UKPDS 90^27^ |
| Woman | -0.30 | NA | Bootstrapped Samples | UKPDS 90^27^ |
| Age at diagnosis | -0.05 | NA | Bootstrapped Samples | UKPDS 90^27^ |
| Smoker last year | 2.02 | NA | Bootstrapped Samples | UKPDS 90^27^ |
| Smoker at diagnosis | 5.54 | NA | Bootstrapped Samples | UKPDS 90^27^ |
| Diabetes Duration | -1.57 | NA | Bootstrapped Samples | UKPDS 90^27^ |
| Time to developing Micro or Macro Albuminuria – Weibull time to event equation with time varying covariates | | | | |
| Shape | 1.14 | NA | Bootstrapped Samples | UKPDS 90^27^ |
| Constant | -9.05 | NA | Bootstrapped Samples | UKPDS 90^27^ |
| Woman | -0.46 | NA | Bootstrapped Samples | UKPDS 90^27^ |
| Age at diagnosis | 0.01 | NA | Bootstrapped Samples | UKPDS 90^27^ |
| Smoking status | 0.33 | NA | Bootstrapped Samples | UKPDS 90^27^ |
| SBP / 10 | 0.19 | NA | Bootstrapped Samples | UKPDS 90^27^ |
| HbA1c | 0.17 | NA | Bootstrapped Samples | UKPDS 90^27^ |
| BMI | 0.03 | NA | Bootstrapped Samples | UKPDS 90^27^ |
| HDL * 10 | -0.03 | NA | Bootstrapped Samples | UKPDS 90^27^ |
| Time to developing peripheral vascular disease – Weibull time to event model with time varying covariates | | | | |
| Shape | 1.52 | NA | Bootstrapped Samples | UKPDS 90^27^ |
| Constant | -12.27 | NA | Bootstrapped Samples | UKPDS 90^27^ |
| Age at diagnosis | 0.06 | NA | Bootstrapped Samples | UKPDS 90^27^ |
| Smoking Status | 0.87 | NA | Bootstrapped Samples | UKPDS 90^27^ |
| SBP /10 | 0.10 | NA | Bootstrapped Samples | UKPDS 90^27^ |
| HbA1c | 0.09 | NA | Bootstrapped Samples | UKPDS 90^27^ |
| BMI | 0.02 | NA | Bootstrapped Samples | UKPDS 90^27^ |
| LDL * 10 | 0.02 | NA | Bootstrapped Samples | UKPDS 90^27^ |
| Time to developing atrial fibrillation – Exponential time to event model with time varying covariates | | | | |
| Constant | -13.31 | NA | Bootstrapped Samples | UKPDS 90^27^ |
| Age at diagnosis | 0.09 | NA | Bootstrapped Samples | UKPDS 90^27^ |
| BMI | 0.06 | NA | Bootstrapped Samples | UKPDS 90^27^ |
| Is eGFR below 60 mL/min/1.73m^2^ – Weibull time to event model with time varying covariates | | | | |
| Shape | 1.87 | NA | Bootstrapped Samples | UKPDS 90^27^ |
| Constant | -11.78 | NA | Bootstrapped Samples | UKPDS 90^27^ |
| Woman | 0.74 | NA | Bootstrapped Samples | UKPDS 90^27^ |
| Afro Caribbean Ethnicity | -0.97 | NA | Bootstrapped Samples | UKPDS 90^27^ |
| Indian Ethnicity | -0.30 | NA | Bootstrapped Samples | UKPDS 90^27^ |
| Age at diagnosis | 0.08 | NA | Bootstrapped Samples | UKPDS 90^27^ |
| SBP / 10 | 0.08 | NA | Bootstrapped Samples | UKPDS 90^27^ |
| BMI | 0.01 | NA | Bootstrapped Samples | UKPDS 90^27^ |
| LDL * 10 | -0.03 | NA | Bootstrapped Samples | UKPDS 90^27^ |
| HDL * 10 | 0.01 | NA | Bootstrapped Samples | UKPDS 90^27^ |
| eGFR if someone’s eGFR is over 60 mL/min/1.73m^2^ – tobit regression | | | | |
| Sigma | 12.57 | NA | Fixed | UKPDS 90^27^ |
| Constant | 23.97 | NA | Bootstrapped Samples | UKPDS 90^27^ |
| Woman | -2.99 | NA | Bootstrapped Samples | UKPDS 90^27^ |
| Afro Caribbean Ethnicity | 3.42 | NA | Bootstrapped Samples | UKPDS 90^27^ |
| Indian Ethnicity | 2.40 | NA | Bootstrapped Samples | UKPDS 90^27^ |
| eGFR | 0.41 | NA | Bootstrapped Samples | UKPDS 90^27^ |
| eGFR at diagnosis | 0.30 | NA | Bootstrapped Samples | UKPDS 90^27^ |
| Diabetes duration | -3.01 | NA | Bootstrapped Samples | UKPDS 90^27^ |
| eGFR if someone’s eGFR is below 60 mL/min/1.73m^2^ – tobit regression | | | | |
| Sigma | 9.45 | NA | Fixed | UKPDS 90^27^ |
| Constant | 26.10 | NA | Bootstrapped Samples | UKPDS 90^27^ |
| Woman | -2.41 | NA | Bootstrapped Samples | UKPDS 90^27^ |
| Afro Caribbean Ethnicity | 2.16 | NA | Bootstrapped Samples | UKPDS 90^27^ |
| Indian Ethnicity | 1.23 | NA | Bootstrapped Samples | UKPDS 90^27^ |
| eGFR | 0.57 | NA | Bootstrapped Samples | UKPDS 90^27^ |
| eGFR at diagnosis | 0.14 | NA | Bootstrapped Samples | UKPDS 90^27^ |
| Diabetes duration | -3.28 | NA | Bootstrapped Samples | UKPDS 90^27^ |
| NA, not applicable; UKPDS, united kingdom prospective diabetes study; BMI, body mass index; LDL, low density lipoprotein; HDL, high density lipoprotein; SBP, systolic blood pressure; WBC, white blood cell count; eGFR, Estimated Glomerular Filtration Rate | | | | |

# Health Economics and Decision Modelling Analysis Plan

Double click on the icon above to the open the analysis plan

# The probability of having different events in the model after 10 years

|  | **Control** | **Intervention** | **Difference** |
| --- | --- | --- | --- |
| 1st MI | 17.64% | 17.62% | -0.02% |
| 2nd MI | 1.21% | 1.21% | 0.00% |
| 1st Stroke | 9.53% | 9.51% | -0.02% |
| 2nd Stroke | 1.60% | 1.60% | 0.00% |
| CHF | 9.91% | 9.89% | -0.02% |
| IHD | 12.93% | 12.93% | 0.00% |
| Blindness | 7.22% | 7.21% | -0.02% |
| Ulcer | 6.30% | 6.27% | -0.02% |
| 1st Amputation | 5.44% | 5.42% | -0.01% |
| 2nd Amputation | 1.22% | 1.22% | 0.00% |
| Renal Failure | 5.52% | 5.52% | 0.00% |
| PVD | 16.97% | 16.93% | -0.03% |
| Micro or Macro Albuminuria | 36.38% | 36.31% | -0.07% |
| Atrial Fibrillation | 3.01% | 3.00% | -0.01% |
| Breast Cancer | 0.57% | 0.56% | 0.00% |
| Colorectal Cancer | 1.00% | 1.00% | 0.00% |
| Depression | 45.63% | 45.63% | 0.00% |
| Osteoarthritis | 16.17% | 16.17% | 0.00% |
| Abbreviations: MI, myocardial infarction; CHF, chronic heart failure; IHD, ischaemic heart disease, PVD, peripheral vascular disease | | | |

# References

1. Curtis L, Burns, A. Unit Costs of Health and Social Care 2020. Canterbury: University of Kent 2020.

2. Gillett M, Dallosso HM, Dixon S, et al. Delivering the diabetes education and self management for ongoing and newly diagnosed (DESMOND) programme for people with newly diagnosed type 2 diabetes: cost effectiveness analysis. *Bmj-Brit Med J* 2010;341 doi: ARTN c4093

10.1136/bmj.c4093

3. Sheffield Type 2 Diabetes Treatment Model version 3: with the Embedding RCT health economic analyses implemented [program]. 1 version, 2023.

4. Davies MJ, Bodicoat DH, Brennan A, et al. Uptake of self-management education programmes for people with type 2 diabetes in primary care through the embedding package: a cluster randomised control trial and ethnographic study. *BMC Prim Care* 2024;25(1):136. doi: 10.1186/s12875-024-02372-x [published Online First: 2024/04/26]

5. Sargeant JA, Brady EM, Zaccardi F, et al. Adults with early-onset type 2 diabetes (aged 18-39 years) are severely underrepresented in diabetes clinical research trials. *Diabetologia* 2020;63(8):1516-20. doi: 10.1007/s00125-020-05174-9 [published Online First: 2020/06/03]

6. Davies M, Agarwal S, Bodicoat DH, et al. Increasing uptake of self-management education programmes for type 2 diabetes in primary care: the Embedding research programme including an RCT. *Programme Grants for Applied Research;* **In Press**

7. Sone H, Katagiri A, Ishibashi S, et al. Effects of lifestyle modifications on patients with type 2 diabetes: the Japan Diabetes Complications Study (JDCS) study design, baseline analysis and three year-interim report. *Horm Metab Res* 2002;34(9):509-15. doi: 10.1055/s-2002-34791 [published Online First: 2002/10/18]

8. Young RJ, Taylor J, Friede T, et al. Pro-active call center treatment support (PACCTS) to improve glucose control in type 2 diabetes: a randomized controlled trial. *Diabetes Care* 2005;28(2):278-82. doi: 10.2337/diacare.28.2.278 [published Online First: 2005/01/29]

9. Deakin TA, Cade JE, Williams R, et al. Structured patient education: the diabetes X-PERT Programme makes a difference. *Diabet Med* 2006;23(9):944-54. doi: 10.1111/j.1464-5491.2006.01906.x [published Online First: 2006/08/23]

10. Adolfsson ET, Walker-Engstrom ML, Smide B, et al. Patient education in type 2 diabetes: a randomized controlled 1-year follow-up study. *Diabetes Res Clin Pract* 2007;76(3):341-50. doi: 10.1016/j.diabres.2006.09.018 [published Online First: 2006/10/31]

11. Ko SH, Song KH, Kim SR, et al. Long-term effects of a structured intensive diabetes education programme (SIDEP) in patients with Type 2 diabetes mellitus--a 4-year follow-up study. *Diabet Med* 2007;24(1):55-62. doi: 10.1111/j.1464-5491.2007.02013.x [published Online First: 2007/01/18]

12. Davies MJ, Heller S, Skinner TC, et al. Effectiveness of the diabetes education and self management for ongoing and newly diagnosed (DESMOND) programme for people with newly diagnosed type 2 diabetes: cluster randomised controlled trial. *BMJ* 2008;336(7642):491-5. doi: 10.1136/bmj.39474.922025.BE [published Online First: 2008/02/16]

13. Sturt JA, Whitlock S, Fox C, et al. Effects of the Diabetes Manual 1:1 structured education in primary care. *Diabet Med* 2008;25(6):722-31. doi: 10.1111/j.1464-5491.2008.02451.x [published Online First: 2008/04/26]

14. Gary TL, Batts-Turner M, Yeh HC, et al. The effects of a nurse case manager and a community health worker team on diabetic control, emergency department visits, and hospitalizations among urban African Americans with type 2 diabetes mellitus: a randomized controlled trial. *Arch Intern Med* 2009;169(19):1788-94. doi: 10.1001/archinternmed.2009.338 [published Online First: 2009/10/28]

15. Trento M, Gamba S, Gentile L, et al. Rethink Organization to iMprove Education and Outcomes (ROMEO): a multicenter randomized trial of lifestyle intervention by group care to manage type 2 diabetes. *Diabetes Care* 2010;33(4):745-7. doi: 10.2337/dc09-2024 [published Online First: 2010/01/28]

16. Walker EA, Shmukler C, Ullman R, et al. Results of a successful telephonic intervention to improve diabetes control in urban adults: a randomized trial. *Diabetes Care* 2011;34(1):2-7. doi: 10.2337/dc10-1005 [published Online First: 2011/01/05]

17. Ali MK, Singh K, Kondal D, et al. Effectiveness of a Multicomponent Quality Improvement Strategy to Improve Achievement of Diabetes Care Goals: A Randomized, Controlled Trial. *Ann Intern Med* 2016;165(6):399-408. doi: 10.7326/M15-2807 [published Online First: 2016/07/12]

18. Odnoletkova I, Ramaekers D, Nobels F, et al. Delivering Diabetes Education through NurseLed Telecoaching. Cost-Effectiveness Analysis. *Plos One* 2016;11(10) doi: 10.1371/journal.pone.0163997

19. Andrews RC, Cooper AR, Montgomery AA, et al. Diet or diet plus physical activity versus usual care in patients with newly diagnosed type 2 diabetes: the Early ACTID randomised controlled trial. *Lancet* 2011;378(9786):129-39. doi: 10.1016/S0140-6736(11)60442-X [published Online First: 2011/06/28]

20. Hayes A, Arima H, Woodward M, et al. Changes in Quality of Life Associated with Complications of Diabetes: Results from the ADVANCE Study. *Value in Health* 2016;19(1):36-41. doi: 10.1016/j.jval.2015.10.010

21. Ara R, Brazier JE. Populating an economic model with health state utility values: moving toward better practice. *Value Health* 2010;13(5):509-18. doi: 10.1111/j.1524-4733.2010.00700.x [published Online First: 2010/03/17]

22. Laxy M, Becker J, Kaehm K, et al. Utility Decrements Associated With Diabetes and Related Complications: Estimates From a Population-Based Study in Germany. *Value in Health* 2021;24(2):274-80. doi: 10.1016/j.jval.2020.09.017

23. Alva M, Gray A, Mihaylova B, et al. THE EFFECT OF DIABETES COMPLICATIONS ON HEALTH-RELATED QUALITY OF LIFE: THE IMPORTANCE OF LONGITUDINAL DATA TO ADDRESS PATIENT HETEROGENEITY. *Health Economics* 2014;23(4):487-500. doi: 10.1002/hec.2930

24. Bagust A, Beale S. Modelling EuroQol health-related utility values for diabetic complications from CODE-2 data. *Health Econ* 2005;14(3):217-30. doi: 10.1002/hec.910 [published Online First: 2004/09/24]

25. Breeze PR, Thomas C, Squires H, et al. School for Public Health Research (SPHR) Diabetes Prevention Model: Detailed Description of Model Background, Methods, Assumptions and Parameters. *HEDS discussion paper No1501* 2015

26. Hayes AJ, Leal J, Gray AM, et al. UKPDS outcomes model 2: a new version of a model to simulate lifetime health outcomes of patients with type 2 diabetes mellitus using data from the 30 year United Kingdom Prospective Diabetes Study: UKPDS 82. *Diabetologia* 2013;56(9):1925-33. doi: 10.1007/s00125-013-2940-y [published Online First: 2013/06/25]

27. Leal J, Alva M, Gregory V, et al. Estimating risk factor progression equations for the UKPDS Outcomes Model 2 (UKPDS 90). *Diabet Med* 2021;38(10):e14656. doi: 10.1111/dme.14656 [published Online First: 2021/07/24]
