# Supplementary material for: A model based cost-utility analysis of Embedding referral to structured self-management education into standard practice (Embedding) compared to usual care for people with type 2 diabetes diagnosis in the last 12 months in England
Source: BMJ Open. 2025 Feb 11;15(2):e093327. doi: 10.1136/bmjopen-2024-093327 (PMC11815453; doi:10.1136/bmjopen-2024-093327)
Supplement: online supplemental file 2 [file bmjopen-15-2-s002.pdf]

HEDMAP – Modelling Sections

This part of the Health Economics and Decision Modelling Analysis Plan (HEDMAP), details our planned analyses of the Embedding Step Wedge randomised controlled trial (RCT). This document expands upon the published protocol.(1)

Population

We will obtain individual characteristics from the baseline characteristics of the Embedding RCT. Where these data are not available, we will obtain patient characteristics from the simulation population reported in NICE NG28.(2) In the event that these data are not available, we will use data from NICE NG 28 alone.(2)

Intervention

Embedding intervention

Control

No embedding

Model

We will use the Sheffield Type 2 Diabetes treatment model version 2.

Perspective

The perspective will be in line with the National Institute for Health and Care Excellence (NICE) 2013 methods guide, in that it will take an NHS and personal social services perspective.(3)

Discounting

Discounting will be in line with NICE's 2013 methods guide with future costs and QALYs discounted at 3.5% per annum.(3)

Costs

Health State Costs

Health state costs will be obtained from the literature, largely from Alva et al 2015.(4)

Intervention costs

These will consist of two components

1) The cost of Embedding itself
2) The cost of SE courses

The methods used to estimate the cost of Embedding itself are detailed elsewhere. So, we do not go into detail here. However, we will convert these costs into a per patient cost for the economic analyses.

The cost of SE courses will be obtained from Gillett et al 2010, assuming that all courses have the same cost as DESMOND.(5)

Prices

All costs will be in 2019/20 prices. Costs from previous years, will be inflated using the HCHS pay and prices index (up until 2014/15) and NHSCII pay and prices index (from 2015/16 onwards).(6, 7)

Effects

Post-Embedding Effects

In the base case analysis, we will apply the mean effects observed in the Embedding study from the statistical analysis of the main Embedding Step Wedge trial. In the base case, we will use the primary endpoint (HbA1c) and any statistically significant secondary endpoints (BMI, SBP, LDL and HDL) to estimate the effect of Embedding. The proportion of people attending structured education (SE) courses will be taken from the Embedding Step Wedge trial. As the step wedge design does not specifically attribute time points to analyses, as a traditional RCT would do, we will assume that these effects from the main study occur at one-year post-embedding.

A sustainability analysis was done on the effect of Embedding on HbA1c and Attendance at SE courses with an additional year of follow up data. We will use these data to estimate the effects of Embedding at two years post course. For items not re-estimated in the sustainability analyses, we will assume that these remain at their year 1 levels at year 2.

Duration of effects

In the long run, many SE programmes have shown long term effects.(8, 9) We will seek expert opinion from Embedding TMG members on the modelled duration of effect.

Scenario analyses

In the base case all effects will be fully maintained for a lifetime.

We will conduct a range of scenario analyses, which will include:

1. In line with suggested changes in NICE guidance, we will discount future costs and QALYs at 1.5%.
2. We will include all outcomes (HbA1c, BMI, SBP, LDL and HDL cholesterol), regardless of statistical significance.
3. We will estimate the proportion of patients in each arm of embedding that attended a SE course in the main study (from Embedding) and give every patient any effects on biomarkers (HbA1c, BMI, SBP, LDL and HDL) that are statistically significant in a meta-analysis of prominent trials of Structured Education, with the trails in the meta-analysis obtained from a recent review (Sargent et al 2020).(10)
4. We will estimate the proportion of patients in each arm of embedding that attended a SE course (from Embedding) and give every patient any effects (regardless of statistical significance) in a meta-analysis of prominent trials of Structured Education.
5. We will estimate the proportion of patients in each arm of embedding that attended a SE course in the sustainability analyses and give every patient any effects that are statistically significant in a meta-analysis of prominent trials of Structured Education.
6. We will conduct a variety of scenario analyses on the duration of any effect of SE. These will be:
    a. 10 years where the effect is fully maintained, and no effect afterwards
    b. 10 years full maintenance of effect, with a gradual decline until 21 years (Steno II trial long term Analyses(9)).

Outcome measures

The primary outcome measure will be the incremental cost-effectiveness ratio (ICER). This is calculated by:

Difference in cost between intervention and control / Differences in quality adjusted life years between intervention and control

Unless one of intervention or control is more effective and cheaper than the other, in which case an ICER is not calculated, and the more effective and cheaper intervention is cost-effective.

We will compare the ICER with the lower end of NICE's typical ICER range, £20,000 per QALY gained.(3)

In line with our protocol, we will produce cost-effectiveness acceptability curves and conduct value of information analyses using Sheffield Accelerated Value of Information methods. Which will produce the total value of information and the value of information relating to individual model parameters.(11) We will at a minimum produce estimates for the following parameter combinations: all effectiveness parameters, all cost parameters, all utility parameters.

As the SPHR type 2 diabetes policy model can undertake probabilistic analyses in parallel, meaning that results can be produced efficiently (5,000 patients and 1,000 PSA runs takes about 30 minutes to run). We will do all scenario analyses as Probabilistic Analyses, as the model is non-linear therefore the deterministic results (using mean estimates of all model parameters) should not be believed.

Discrepancies from the protocol

We will use the SPHR Type 2 Diabetes Treatment Model version 2, rather than the Sheffield Type 2 Diabetes Policy Model as this model has been updated from the Sheffield Type 2 Diabetes Policy Model.

All analyses will be probabilistic analyses, rather than some scenarios being deterministic.

References

1. Davies MJ, Kristunas CA, Alshreef A, Dixon S, Eborall H, Glab A, et al. The impact of an intervention to increase uptake to structured self-management education for people with type 2 diabetes mellitus in primary care (the embedding package), compared to usual care, on glycaemic control: study protocol for a mixed methods study incorporating a wait-list cluster randomised controlled trial. BMC Fam Pract. 2019;20(1):152.
2. National Institute for Health and Care Excellence. Type 2 diabetes in adults: management. 2015. Contract No.: 24th January 2022.
3. National Institute for Health and Care Excellence. Guide to the methods of technology appraisal 2013. London: National Institute for Health and Care Excellence,; 2013.
4. Alva ML, Gray A, Mihaylova B, Leal J, Holman RR. The impact of diabetes-related complications on healthcare costs: new results from the UKPDS (UKPDS 84). Diabet Med. 2015;32(4):459-66.
5. Gillett M, Dallosso HM, Dixon S, Brennan A, Carey ME, Campbell MJ, et al. Delivering the diabetes education and self management for ongoing and newly diagnosed (DESMOND) programme for people with newly diagnosed type 2 diabetes: cost effectiveness analysis. BMJ. 2010;341:c4093.
6. Curtis L. Unit Costs of Health and Social Care. Personal Social Services Research Unit, University of Kent, Canterbury; 2010.
7. Curtis L, Burns A. Unit Costs of Health and Social Care 2020. Personal Social Services Research Unit, University of Kent, Canterbury; 2020.
8. Khunti K, Gray LJ, Skinner T, Carey ME, Realf K, Dallosso H, et al. Effectiveness of a diabetes education and self management programme (DESMOND) for people with newly diagnosed type 2 diabetes mellitus: three year follow-up of a cluster randomised controlled trial in primary care. BMJ. 2012;344:e2333.
9. Gaede P, Oellgaard J, Carstensen B, Rossing P, Lund-Andersen H, Parving HH, et al. Years of life gained by multifactorial intervention in patients with type 2 diabetes mellitus and microalbuminuria: 21 years follow-up on the Steno-2 randomised trial. Diabetologia. 2016;59(11):2298-307.
10. Sargeant JA, Brady EM, Zaccardi F, Tippins F, Webb DR, Aroda VR, et al. Adults with early-onset type 2 diabetes (aged 18-39 years) are severely underrepresented in diabetes clinical research trials. Diabetologia. 2020;63(8):1516-20.
11. Strong M, Oakley JE, Brennan A. Estimating multiparameter partial expected value of perfect information from a probabilistic sensitivity analysis sample: a nonparametric regression approach. Med Decis Making. 2014;34(3):311-26.
