# Supplementary material for: A model based cost-utility analysis of Embedding referral to structured self-management education into standard practice (Embedding) compared to usual care for people with type 2 diabetes diagnosis in the last 12 months in England
Source: BMJ Open. 2025 Feb 11;15(2):e093327. doi: 10.1136/bmjopen-2024-093327 (PMC11815453; doi:10.1136/bmjopen-2024-093327)
Supplement: Supplementary file 3 [file bmjopen-2024-093327.reviewer_comments.pdf]

PEER REVIEW HISTORY

BMJ Open publishes all reviews undertaken for accepted manuscripts. Reviewers are asked to complete a checklist review form and are provided with free text boxes to elaborate on their assessment. These free text comments are reproduced below.

ARTICLE DETAILS

Title (Provisional)

A model based cost-utility analysis of embedding referral to structured self-management education into standard practice (Embedding) compared to usual care for people with type 2 diabetes diagnosis in the last 12 months in England.

Authors

Pollard, Daniel John; Keetharuth, Anju; Brennan, Alan; Bodicoat, Danielle; Glab, Agnieszka; Hadjiconstantinou, Michelle; Mensa, Joesph; Northern, Alison; Davies, Melanie J

VERSION 1 - REVIEW

**Reviewer**	**1**
**Name**	**Dinneen, Sean**
**Affiliation**	**National University of Ireland, Galway, Department of Medicine**
**Date**	**30-Oct-2024**
**COI**	**None**

Summary

The authors present a cost effectiveness analysis of a strategy to embed a process of referral of patients with type 2 diabetes to one of 4 DSME programmes in approx 66 practices in England. The cost-effectiveness analysis is based on the findings of a cluster RCT the results of which have been previously published. The design of the RCT was complex (cluster randomisation; stepped wedge design and wait-list assignment of practices to intervention or control arm). The complex intervention involved a "marketing strategy" for DSME focused on GP surgeries, providers (of DSME) and commissioners. An online portal containing an embedding toolkit was overseen by 2 "embedders" whose role it was to engage with providers and/or practices to ensure early referral to one of 4 DSME programmes (mainly DESMOND). The delivery of the intervention (in the original RCT) was hampered by COVID lockdowns whereby the embedders (and DSME delivery in general) were re-directed to the pandemic response. The original RCT found statistically (but not clinically) significant differences in the primary outcome (change in HbA1c) in certain sub-groups. However, the

overall trial reported no improvement in referral rates with use of the embedder approach. The health economic analyses are comprehensive and include cost-effectiveness analyses based on the results of the RCT and also modelling based on UKPDS data and a NIHR (Sheffield) health economic model for type 2 diabetes. The overall results showed that the intervention was low cost and likely to be cost effective (in terms of cost per QALY) in most (but not all) of the scenarios tested.

Major comments

While the paper is likely to be of interest to health economists and methodologists it is a hard read for clinicians (and I suspect commissioners) even those (like myself) with an interest in DSME. The latter groups would require more convincing evidence of (potential) benefit before introducing the "embedding tools" into routine clinical practice. The section entitled "Implications for Practice" requires revision to make it more readable (and relevant) to clinicians. The sentence “As such, the implications for practice appear to be driven by two issues, first, the decision maker’s view as to the relative validity of the 12- and 24-month results, and second, their generalisability to post-pandemic organisational and patient behaviour” seems to be side-stepping the issue of declaring whether or not there is evidence to support the embedding approach used in the cRCT. It would appear (to this reviewer) that there is not sufficient evidence to support the approach and (perhaps) it would be better to state that more clearly. If the original trial was hampered by circumstances then so be it. Another attempt at an embedding approach (with learning from the original trial) may be required rather than an unconvincing CE analyses being used to try to sell the approach to clinicians and commissioners.

**Reviewer**	**2**
**Name**	**Li, Mingzi**
**Affiliation**	**Peking University Health Science Centre**
**Date**	**19-Nov-2024**
**COI**	**None**

This is a very interesting paper investing the cost-effectiveness of an Embedding package of promoting SSME for people with T2DM in England. The research question is clear and the design and analyzing of the data are robust. The discussion and conclusion are reasonable.

**Reviewer**	**3**
**Name**	**Kelson, Zoe**
**Affiliation**	**University of Exeter, Mathematics**

**Date**	**15-Dec-2024**
**COI**	**None**

This wait-list cluster randomised controlled trial was undertaken to estimate cost-effectiveness of an implementation package embedding the referral of people with T2DM to SSME from primary care into routine practice.

Reviewer comments:

"All methods are in line with the study protocol, the analysis plan for the modelling is available as a supplementary material."

It appears that the analysis plan has been included as a pdf thumbnail? Can this please be provided in full for review?

"64 practices were randomised"

Can the authors please comment on whether the included practices can be considered to be representative?

Can the ratio of randomisation between wait-list and intervention programme type please be specified here?

Was the randomisation allocation stratified by any variables?

"Four SSME programmes were used: DESMOND (https://www.desmond.nhs.uk/), Diabetes 2gether/Diabetes 4ward (https://www.oxfordhealth.nhs.uk/community-diabetes/education/), Spotlight (Lincolnshire Community Health Services NHS Trust, terminated) and Xpert Health (https://www.xperthealth.org.uk/)."

Can the authors please consider exploring subgroup analyses by type of programme used?

"As this was a feasibility study, there was no formal sample size calculation. The number of practices was based on a balance between practicalities and having sufficient data to assess its quality and to give adequate variation in urban and rural practices..." [Davies et. al]

Can the authors please discuss sample size, declaring study power considerations, in the main article?

"Long-term sequelae of T2DM based on the values for HbA1c, BMI, HDL, LDL and age/sex were estimated using the NIHR School for Public Health Research T2DM treatment model version 3.0"

and

"As the model has been shown to be nonlinear, the means were calculated via the probabilistic sensitivity analysis (PSA). Bootstrapped trial estimates were plotted on a cost-effectiveness plane and the associated cost-effectiveness acceptability curves generated"

Appropriate modelling methods have been applied by the authors.

Can more detail on the model formulation please be provided in the supplementary material?

"PSA was undertaken with all model parameters being randomly sampled from their associated distributions. Using a threshold ICER of £20,000 per QALY gained, we established that simulating 2,000 samples of 5,000 patients (10 million simulations in total) was sufficient to generate robust estimates of uncertainty in cost and QALY differences"

This probabilistic sensitivity analysis on parameter values helps to quantify and communicate uncertainty in the model outputs. The model parameters, with distribution and source, have been suitably tabulated in the supplementary material.

"Alongside our base case, six further scenario analyses were also undertaken to assess the impact of uncertainties not captured by probability distributions of the individual parameters."

A number of insightful what-if scenarios have been explored.

Can the Tables please report uncertainty intervals for all point estimates?

"Strengths and weaknesses"

The authors provide a thorough discussion on the study limitations.

Thanks for providing a copy of the CHEERS checklist.

VERSION 1 - AUTHOR RESPONSE

**Reviewer: 1**<br>**Dr. Sean Dinneen, National University of Ireland, Galway**	
**Reviewer Comment**	**Author's Response**
Summary<br>The authors present a cost effectiveness analysis of a strategy to embed a process of referral of patients with type 2 diabetes to one of 4 DSME programmes in approx 66 practices in England. The cost-effectiveness analysis is based on the findings of a cluster RCT the results of which have been previously published. The design of the RCT was complex (cluster randomisation; stepped wedge design and wait-list assignment of practices to intervention or control arm). The complex intervention involved a "marketing strategy" for DSME focused on GP surgeries, providers (of DSME) and commissioners. An online portal containing an embedding toolkit was overseen by 2 "embedders" whose role it was to engage with providers and/or practices to ensure early referral to one of 4 DSME programmes (mainly DESMOND). The delivery of the intervention (in the original RCT) was hampered by COVID lockdowns whereby the embedders (and DSME delivery in general) were re-directed to the pandemic response. The original RCT found statistically (but not clinically) significant differences in the primary outcome (change in HbA1c) in certain sub-groups. However, the overall	Thank you for taking the time to read our paper.

<table>
<tr><td>trial reported no improvement in referral rates with use of the embedder approach. The health economic analyses are comprehensive and include cost-effectiveness analyses based on the results of the RCT and also modelling based on UKPDS data and a NIHR (Sheffield) health economic model for type 2 diabetes. The overall results showed that the intervention was low cost and likely to be cost effective (in terms of cost per QALY) in most (but not all) of the scenarios tested.</td><td></td></tr>
<tr><td>While the paper is likely to be of interest to health economists and methodologists it is a hard read for clinicians (and I suspect commissioners) even those (like myself) with an interest in DSME. The latter groups would require more convincing evidence of (potential) benefit before introducing the "embedding tools" into routine clinical practice.</td><td rowspan="6">We agree with these points and have significantly edited edited the implications for practice section of the discussion to reflect these issues. We also edited the conclusions section of the abstract to reflect these points.</td></tr>
<tr><td>The section entitled "Implications for Practice" requires revision to make it more readable (and relevant) to clinicians.</td></tr>
<tr><td>The sentence “As such, the implications for practice appear to be driven by two issues, first, the decision maker’s view as to the relative validity of the 12- and 24-month results, and second, their generalisability to post-pandemic organisational and patient behaviour” seems to be side-stepping the issue of declaring whether or not there is evidence to support the embedding approach used in the cRCT.</td></tr>
<tr><td>It would appear (to this reviewer) that there is not sufficient evidence to support the approach and (perhaps) it would be better to state that more clearly. If the original trial was hampered by circumstances then so be it.</td></tr>
<tr><td>Another attempt at an embedding approach (with learning from the original trial) may be required rather than an unconvincing CE analyses being used to try to sell the approach to clinicians and commissioners.</td></tr>
<tr><td colspan="2">Reviewer: 2 Dr. Mingzi Li, Peking University Health Science Centre</td></tr>
<tr><td>Reviewer Comment</td><td>Author’s Response</td></tr>
<tr><td>This is a very interesting paper investing the cost-effectiveness of an Embedding package of promoting SSME for people with T2DM in England. The research question is clear and the design and analyzing of the data are robust. The discussion and conclusion are reasonable.</td><td>Thank you for taking the time to read our paper</td></tr>
<tr><td colspan="2">Reviewer: 3. Prof. Zoe Kelson, University of Exeter</td></tr>
<tr><td>"All methods are in line with the study protocol, the analysis plan for the modelling is available as a supplementary material." It appears that the analysis plan has been included as a pdf thumbnail? Can this please be provided in full for review?</td><td>Thank you for taking the time to read our paper.

Sorry about this issue.

The HEDMAP is an embedding pdf in the word document. It opens up by double clicking on it when you have downloaded the word document which we believe the supplementary</td></tr>
</table>

	material will be formatted as in this journal.<br><br>In the revision we have added the pdf of the analysis plan as a reviewer specific supplementary material.
"64 practices were randomised"<br>Can the authors please comment on whether the included practices can be considered to be representative?	We have added a brief summary of the statements on representativeness from the main trial paper and the protocol paper to line 126 in the clean manuscript.
Can the ratio of randomisation between wait-list and intervention programme type please be specified here? Was the randomisation allocation stratified by any variables?	Brief details have been added to our paper on lines 127-133 (clean manuscript) on the randomisation, which summarises the main trial paper. There is an explicit reference to the main trial results paper for further details.
"Four SSME programmes were used: DESMOND (https://www.desmond.nhs.uk/), Diabetes 2gether/Diabetes 4ward (https://www.oxfordhealth.nhs.uk/community-diabetes/education/), Spotlight (Lincolnshire Community Health Services NHS Trust, terminated) and Xpert Health (https://www.xperthealth.org.uk/)."<br>Can the authors please consider exploring subgroup analyses by type of programme used?	We were able to conduct sensitivity around one-year HbA1c for the type of course offered by providers. We have added additional scenario analyses using these estimates of one-year HbA1c to our economic modelling results. We have also added the other scenarios around one-year HbA1c presented in Davies *et al* as scenarios in the economic modelling. For the scenarios in the white ethnic group, other ethnic groups and baseline HbA1c $\geq$ 47.5 mmol/mol we have also changed the simulated population in our economic model to reflect these subgroups.<br><br>We have rerun all analyses to make sure that the same version of R (version 4.4.1) is being used to conduct all analyses in the manuscript.
"As this was a feasibility study, there was no formal sample size calculation. The number of practices was based on a balance between practicalities and having sufficient data to assess its quality and to give adequate variation in urban and rural	The sample size is discussed in the main results paper and we do not think it is appropriate to

practices..." [Davies et. al]<br><br>Can the authors please discuss sample size, declaring study power considerations, in the main article?	add additional details to the health economic paper.<br><br>Brief details of this have been added to lines 127-133 (clean manuscript) in the track changed revision.
"Long-term sequelae of T2DM based on the values for HbA1c, BMI, HDL, LDL and age/sex were estimated using the NIHR School for Public Health Research T2DM treatment model version 3.0"<br>and<br>"As the model has been shown to be nonlinear, the means were calculated via the probabilistic sensitivity analysis (PSA). Bootstrapped trial estimates were plotted on a cost-effectiveness plane and the associated cost-effectiveness acceptability curves generated"<br>Appropriate modelling methods have been applied by the authors.<br>Can more detail on the model formulation please be provided in the supplementary material?	A full set of documentation is already provided within our open-source code.<br><br>We have attached this as editor only information so this can be distributed to you efficiently.<br><br>We have put added to this Github, and this should be available via the DOI in the manuscript soon, as it is waiting sign off from the library.
"PSA was undertaken with all model parameters being randomly sampled from their associated distributions. Using a threshold ICER of £20,000 per QALY gained, we established that simulating 2,000 samples of 5,000 patients (10 million simulations in total) was sufficient to generate robust estimates of uncertainty in cost and QALY differences"<br>This probabilistic sensitivity analysis on parameter values helps to quantify and communicate uncertainty in the model outputs. The model parameters, with distribution and source, have been suitably tabulated in the supplementary material.	No response needed
"Alongside our base case, six further scenario analyses were also undertaken to assess the impact of uncertainties not captured by probability distributions of the individual parameters."<br>A number of insightful what-if scenarios have been explored.	No response needed
"Strengths and weaknesses"<br>The authors provide a thorough discussion on the study limitations.<br><br>Thanks for providing a copy of the CHEERS checklist.	No response needed

VERSION 2 - REVIEW

Reviewer 1

Name **Dinneen, Sean**

Affiliation **National University of Ireland, Galway, Department of Medicine**

Date **20-Jan-2025**

COI

The authors have addressed my concerns and the changes to the manuscript enhance the overall messages for clinicians and commissioners
